# Components of the interleukin-33/ST2 system are differentially expressed and regulated in human cardiac cells and in cells of the cardiac vasculature

**DOI:** 10.1016/j.yjmcc.2013.03.020

**Published:** 2013-07

**Authors:** Svitlana Demyanets, Christoph Kaun, Richard Pentz, Konstantin A. Krychtiuk, Sabine Rauscher, Stefan Pfaffenberger, Andreas Zuckermann, Arezu Aliabadi, Marion Gröger, Gerald Maurer, Kurt Huber, Johann Wojta

**Affiliations:** a Department of Internal Medicine II, Medical University of Vienna, Waehringer-Guertel 18-20, 1090 Vienna, Austria; b Department of Laboratory Medicine, Medical University of Vienna, Waehringer-Guertel 18-20, 1090 Vienna, Austria; c Ludwig Boltzmann Cluster for Cardiovascular Research, Waehringer-Guertel 18-20, 1090 Vienna, Austria; d Skin and Endothelium Research Division (SERD), Department of Dermatology, Medical University of Vienna, Waehringer-Guertel 18-20, 1090 Vienna, Austria; e Core Facility Imaging, Medical University of Vienna, Waehringer-Guertel 18-20, 1090 Vienna, Austria; f Department of Surgery, Medical University of Vienna, Waehringer-Guertel 18-20, 1090 Vienna, Austria; g 3rd Medical Department for Cardiology and Emergency Medicine, Wilhelminenhospital, Montleartstraße 37, 1160 Vienna, Austria

**Keywords:** Interleukin-33, ST2, Cardiac fibroblasts, Cardiac myocytes, Cytokines

## Abstract

Interleukin-33 (IL-33) is a recently described member of the IL-1 family of cytokines, which was identified as a ligand for the ST2 receptor. Components of the IL-33/ST2 system were shown to be expressed in normal and pressure overloaded human myocardium, and soluble ST2 (sST2) has emerged as a prognostic biomarker in myocardial infarction and heart failure. However, expression and regulation of IL-33 in human adult cardiac myocytes and fibroblasts was not tested before. In this study we found that primary human adult cardiac fibroblasts (HACF) and human adult cardiac myocytes (HACM) constitutively express nuclear IL-33 that is released during cell necrosis. Tumor necrosis factor (TNF)-α, interferon (IFN)-γ and IL-1β significantly increased both IL-33 protein and IL-33 mRNA expression in HACF and HACM as well as in human coronary artery smooth muscle cells (HCASMC). The nuclear factor-κB (NF-κB) inhibitor dimethylfumarate inhibited TNF-α- and IL-1β-induced IL-33 production as well as nuclear translocation of p50 and p65 NF-κB subunits in these cells. Mitogen-activated protein/extracellular signal-regulated kinase inhibitor U0126 abrogated TNF-α-, IFN-γ-, and IL-1β-induced and Janus-activated kinase inhibitor I reduced IFN-γ-induced IL-33 production. We detected IL-33 mRNA in human myocardial tissue from patients undergoing heart transplantation (n = 27) where IL-33 mRNA levels statistically significant correlated with IFN-γ (r = 0.591, p = 0.001) and TNF-α (r = 0.408, p = 0.035) mRNA expression. Endothelial cells in human heart expressed IL-33 as well as ST2 protein. We also reveal that human cardiac and vascular cells have different distribution patterns of ST2 isoforms (sST2 and transmembrane ST2L) mRNA expression and produce different amounts of sST2 protein. Both human macrovascular (aortic and coronary artery) and heart microvascular endothelial cells express specific mRNA for both ST2 isoforms (ST2L and sST2) and are a source for sST2 protein, whereas cardiac myocytes, cardiac fibroblasts and vascular SMC express only minor amounts of ST2 mRNA and do not secrete detectable amounts of sST2 antigen. In accordance with the cellular distribution of ST2 receptor, human cardiac fibroblasts and myocytes as well as HCASMC did not respond to treatment with IL-33, as recombinant human IL-33 did not induce NF-κB p50 and p65 subunits nuclear translocation or increase IL-6, IL-8, and monocyte chemoattractant protein (MCP-1) level in HACF, HACM and HCASMC. In summary, we found that endothelial cells seem to be the source of sST2 and the target for IL-33 in the cardiovascular system. IL-33 is expressed in the nucleus of human adult cardiac fibroblasts and myocytes and released during necrosis. Proinflammatory cytokines TNF-α, IFN-γ and IL-1β increase IL-33 in these cells *in vitro*, and IL-33 mRNA levels correlated with TNF-α and IFN-γ mRNA expression in human myocardial tissue.

## Highlights

• IL-33 is in nucleus of cardiac myocytes, cardiac fibroblasts and smooth muscle cells.
• TNF-α, IFN-γ, IL-1β increase IL-33 expression in these cells.
• IL-33 is released during necrosis by these cells.
• Endothelial cells in myocardial tissue predominantly express IL-33 and ST2.
• Endothelial cells express the IL-33 receptor ST2 and secrete soluble ST2.

## 1 Introduction

Interleukin-33 (IL-33) is a recently described member of the IL-1 family of cytokines, which also includes IL-1α, IL-1β and IL-18 [1], [2]. IL-33 was identified as a ligand for the ST2 receptor, which was known for a long time as an orphan receptor [1]. The transmembrane ST2 (ST2L) and the soluble ST2 (sST2) isoforms arise from a dual promoter system to drive differential mRNA expression [3]. ST2L is necessary for the extracellular effects of IL-33 as IL-33 binds to the receptor complex composed of ST2L and IL-1R accessory protein (IL-1RAcP) [4]. Circulating sST2 has emerged as a prognostic biomarker in patients with myocardial infarction (MI) and heart failure [5], [6], [7].

IL-33 is broadly expressed in many tissues but is more restricted at the level of cell types, and shows a different expression pattern in comparison to IL-1β and IL-18. Although hematopoietic cells seem to be the main source of IL-1β and IL-18, only low levels of human IL-33 mRNA were detected in this cell type [1], [2]. Activated dendritic cells and macrophages are the only haematopoietic cells that show low quantities of human IL-33 mRNA [1]. Endothelial cells seem to be the main cell types expressing IL-33 in different organs and tissues in humans such as lung, colon, kidney, liver, skin [8], [9], brain [10], atherosclerotic plaques [11] or adipose tissue [12]. IL-33 is constitutively expressed in the nucleus of endothelial cells from both small and large blood vessels [8], [9], [13], [14]. In addition to endothelial cells, also epithelial cells, keratinocytes, smooth muscle cells (SMC) or fibroblasts express IL-33 [1], [9], [15], [16]. Although increased levels of IL-33 protein in tissues and in circulation have been reported in several diseases such as asthma, rheumatologic pathologies, inflammatory bowel and skin diseases [5], [15], [17], [18], reports showing cellular release of IL-33 after stimulation are scarce [19], [20], [21], [22]. According to present knowledge, IL-33 is specifically released by necrotic cells but kept intracellular during apoptosis by caspases inactivation [23], [24]. This suggests that IL-33 might function as an alarmin belonging to the larger family of damage-associated molecular pattern (DAMP) molecules [9], [25].

Multiple cell types express transmembrane ST2 on their surface and can respond to IL-33 [26]. ST2 is expressed on T helper 2 (Th2)-related immune cells such as Th2 cells, mast cells, basophils, eosinophils, and recently discovered nuocytes, and involved in the activation of these cells [1], [26], [27]. Thus, IL-33 acts as an inflammatory cytokine in Th2-type immune responses during asthma or atopic dermatitis and seems to be responsible for the host defense against helminth infections [28]. ST2L is also expressed on macrophages, and IL-33 has been found to amplify the alternative activation of macrophages [29]. Also non-haematopoietic cells such as endothelial and epithelial cells, express ST2 splice variants [30], [31], [32]. In human endothelial cells, IL-33 induces inflammatory activation through upregulation of IL-6, IL-8, monocyte chemoattractant protein-1 (MCP-1), vascular cell adhesion molecule-1 (VCAM-1), intercellular adhesion molecule-1 (ICAM-1), endothelial selectin (E-selectin), increases vascular permeability and promotes angiogenesis [11], [30], [33].

The regulation of IL-33 expression seems to differ between cell types. Tumor necrosis factor (TNF)-α, IL-1β, interferon-γ (IFN-γ) or lipopolysaccharide (LPS) are activators of IL-33 expression in lung, dermal or synovial fibroblasts, keratinocytes, airway SMC, pancreatic and hepatic stellate cells, intestinal epithelial cells, central nervous system glia, and adipocytes [1], [15], [18], [19], [34], [35]. In human umbilical artery SMC, IL-33 (referred to in that publication as DVS27) was induced by IL-1α, IL-1β, and IFN-γ, but not by LPS, TNF-α or IL-6 [36]. In neonatal rat cardiac fibroblasts and myocytes, IL-33 expression was upregulated by biomechanical strain, angiotensin II (Ang II) and phorbol 12-myristate 13-acetate (PMA); however, neither TNF-α nor IL-1β induced IL-33 expression in these cells [16]. LPS, but not IL-1β and TNF-α, upregulated IL-33 in monocytes [37]. In contrast, endothelial cells respond to proinflammatory or angiogenic activation by TNF-α and IL-1β or vascular endothelial growth factor (VEGF), respectively, with a downregulation of IL-33 expression [8].

However, a possible role of IL-33 and ST2 in the pathogenesis of cardiovascular diseases is still not well defined. Components of the IL-33/ST2 system were shown to be expressed in normal and pressure overloaded human myocardium and in coronary artery endothelium [38] as well as in rat neonatal cardiac myocytes and fibroblasts [16]. However, expression and possible regulation of IL-33 in human adult cardiac myocytes and fibroblasts has not been studied yet. Therefore, in this study we investigated the cellular origin of IL-33 and ST2 and the regulation of these proteins in human cardiac myocytes and fibroblasts and cells of the cardiac vasculature *in vitro*. In addition we studied the cellular localization of IL-33 and ST2 and a possible correlation between IL-33 mRNA levels and the inflammatory cytokines mRNA levels in human myocardial tissue.

## 2 Materials and methods

### 2.1 Cell culture

Primary human adult cardiac fibroblasts (HACF) and primary human adult cardiac myocytes (HACM) were isolated from ventricular tissue obtained from explanted recipients' hearts after heart transplantation and cultivated as described by our group previously [39]. Briefly, cells were cultured in minimum essential medium (M199, Sigma, St. Louis, MO, USA) containing 20% fetal calf serum (FCS), 100 U/ml penicillin, 100 U/ml streptomycin, 0.25 μg/ml fungizone, and 2 mM L-glutamine (all Cambrex, East Rutherford, NJ, USA) at 37 °C in a humidified atmosphere of 5% CO_2_:95% air. Human coronary artery smooth muscle cells (HCASMC) and human aortic SMC (HASMC) were isolated by the explant technique from pieces of coronary arteries and aortas, respectively, obtained from patients undergoing heart transplantation. Such SMC were cultured and characterized as described [40]. Human coronary artery endothelial cells (HCAEC), human aortic endothelial cells (HAEC) and human umbilical vein endothelial cells (HUVEC) were isolated by mild collagenase treatment, characterized and cultivated as described [11]. Human heart microvascular endothelial cells (HHMECs) were isolated from human hearts of patients undergoing heart transplantation. The study has been reviewed and approved by the Ethic Committee of the Medical University of Vienna, Austria.

### 2.2 Treatment of the cells

HACF, HACM and HCASMC were incubated in M199 (Sigma) containing 0.1% bovine serum albumin (BSA, Sigma) for 24 hours (h) prior to treatment with the respective cytokine. Thereafter, the medium was replaced with fresh M199 containing 0.1% BSA, and recombinant human (rh) TNF-α, rh IL-1β, or rh IFN-γ, all obtained from R&D Systems (Minneapolis, MN, USA), was added at the concentrations indicated for time periods between 3 h and 24 h. Additionally, HACF, HACM and HCASMC were treated with TNF-α, IFN-γ or IL-1β, each at 2000 U/mL for 24 h, and then necrosis was induced by subjecting these cells to 5 cycles of freezing to − 80 °C and thawing at 37 °C as described previously [21], [37], [41]. Furthermore, cells were pre-incubated for 30 minutes (min) with the NF-κB inhibitor dimethylfumarate (DMF) [42] (Sigma) at 100 μM before addition of TNF-α, IL-1β or IFN-γ at the indicated concentrations for further 24 h. Additionally, cells were pre-incubated for 30 min with the mitogen-activated protein/extracellular signal-regulated kinase (MEK) inhibitor U0126 (Promega, Madison, WI, USA) at 10 μM or the janus-activated kinase (JAK) 1 and 2 inhibitor JAK inhibitor I (Calbiochem, Merck, Darmstadt, Germany) at 10 μM before addition of TNF-α, IL-1β or IFN-γ for further 24 h. In another set of experiments, HACF, HACM and HCASMC were incubated with rh IL-33 (R&D Systems) at concentrations of 100, 10 or 1 ng/mL or with rh IL-1β at 10 ng/mL (R&D Systems) for time periods between 3 h and 24 h.

### 2.3 Human tissue

Human heart tissue was obtained from the left ventricle of explanted hearts from 27 patients suffering from cardiomyopathy undergoing heart transplantation. Tissue was stored at − 80 °C for RNA isolation. All human material was obtained and processed according to the recommendations of the hospital's Ethics Committee.

### 2.4 Total RNA purification and cDNA preparation

Cells were treated as described (for IL-33 mRNA determination) or left untreated (for measurement of basal total ST2, ST2L, or sST2 mRNA expression), supernatants were removed and total cellular RNA was isolated using High Pure RNA Isolation Kit (Roche, Basel, Switzerland) according to the manufacturer's instructions. Frozen human myocardial tissue was homogenized using a ball mill (Retsch, Haan, Germany), and mRNA was isolated using High Pure RNA Tissue Kit (Roche). The total RNA amount was measured using NanoDrop (Thermo Scientific, Barrington, IL, USA). Reverse transcription was performed using Transcriptor First Strand cDNA Synthesis Kit (Roche). For PCR of ST2 isoforms, cDNA was additionally eluted with MinElute PCR Purification Kit (QIAGEN GmbH, Hilden, Germany).

### 2.5 RealTime-PCR

RealTime-PCR was performed using LightCycler® TaqMan® Master (Roche) according to the manufacturer's instructions. Primers were designed using the Roche UniversalProbeLibrary Assay Design Centre (http://www.universalprobelibrary.com/). Please see Supplemental Table 1 for detailed information on primers used. The amplification conditions consisted of an initial incubation at 95 °C for 10 min, followed by 45 cycles of 95 °C for 10 s, 63 °C for 20 s and 72 °C for 6 s and a final cooling to 40 °C. Data was analyzed using LightCycler Software Version 3.5 (Roche).

### 2.6 Protein determination

IL-33 protein in cell lysates and cell culture supernatants and sST2 protein in cell culture supernatants was measured by specific enzyme-linked immunosorbent assays (ELISAs) (both from R&D Systems). Cells were permeabilized with PBS containing 0.1% Triton X-100 (Sigma). IL-6, IL-8 and MCP-1 antigen in cell culture supernatants was measured by specific ELISAs using monoclonal antibodies (all from Bender MedSystems, Vienna, Austria).

### 2.7 Nuclear extraction and analysis of NF-κB/DNA binding

HACM, HACF or HCASMC were treated with TNF-α or IL-1β (each at 2000 U/mL) for 60 min with or without pre-incubation for 30 min with DMF 100 μM. Additionally, HACM, HACF or HCASMC were incubated for 15, 30 or 60 min with or without rh IL-33 (R&D Systems) at a concentration of 100 ng/mL. Preparation of nuclear extracts was performed using a Nuclear Extract Kit (Active Motif, Rixensart, Belgium) according to the manufacturer's instructions. Quantification of p50 and p65 NF-κB subunits in nuclear extracts of such treated cells was performed using the ELISA-based TransAM^TM^ NF-κB Family kit (Active Motif) as described previously [43].

### 2.8 Immunofluorescence analysis of IL-33 in cultured cells

HACF, HACM and HCASMC were seeded on Permanox chamber slides (Nunc Inc., Neparville, IL, USA). Confluent monolayers were left untreated or stimulated with rh TNF-α, rh IFN-γ or rh IL-1β, each at 2000 U/mL. After incubation for 24 h the cells were washed with PBS and fixed with 3.7% paraformaldehyde. The cells were washed again and then permeabilized with 0.1% Triton X-100 (Sigma) in PBS for 20 min at room temperature, washed in PBS, and blocked with 5% BSA (Sigma) in PBS for 30 min. Subsequently, cells were washed again and incubated overnight with primary mouse monoclonal anti-IL-33 antibody (clone Nessy-1; Alexia Biochemicals, Enzo Life Sciences AG, Lausen, Switzerland) at 4 °C at a dilution of 1:1000 in diluent solution for primary antibody (DAKO, North America, Inc., CA, USA, catalogue number S3022). The same diluent solution without primary antibody was used as a negative control. After washing, a 1:1000 dilution of Alexa Fluor-488 goat anti-mouse immunoglobulin G (IgG) (Invitrogen-Molecular Probes, Paisley, UK) in diluent solution for secondary antibody (DAKO, catalogue number S0809) was incubated with the cells for 1 h at room temperature in the dark. After final washes, cells were mounted using Vectashield mounting medium with 4′,6-diamidino-2-phenylindole (DAPI; Vector Laboratories Inc., Burlingame, CA, USA) for nuclear counter staining and sealed with nail polish. Fluorescence was assessed by microscopy with a confocal laser scanning microscope (LSM-700; Carl Zeiss, Oberkochen, Germany) with 40 × lens using ZEN 2009 software.

### 2.9 Immunofluorescence analysis of IL-33 and ST2 in explanted hearts

Paraffin-embedded sections were deparaffinised and then boiled for antigen retrieval in citrate buffer (Dako). Sections were blocked with Dako blocking solution for 30 min at room temperature. The following primary antibodies were used: mouse monoclonal anti-IL33 antibody in dilution 1:500 (clone Nessy-1; Enzo Life Sciences); rabbit polyclonal anti-ST2 (IL1RL1) antibody 1:100 (Sigma); goat polyclonal anti-troponin I antibody 1:100 (AbD serotec); rabbit polyclonal anti-Von Willebrand Factor (vWF) antibody 1:500 (Dako); mouse monoclonal anti-smooth muscle actin antibody 1:100 (Dako). Primary antibodies were incubated overnight at 4 °C. After extensive washing in PBS, slides were incubated with secondary antibodies for 1 h at room temperature in the dark. Secondary antibodies were Alexa Fluor-488 rabbit anti-goat IgG (Invitrogen-Molecular Probes), Alexa Fluor-546 goat anti-mouse and goat anti-rabbit IgG and Alexa Fluor-633 goat anti-rabbit IgG. All antibodies were diluted in PBS containing 0.1% Triton X-100. Nuclear counter staining with DAPI (1 μg/ml; Sigma) for 10 min at room temperature was performed. Tissue sections were analyzed with a confocal laser scanning microscope (LSM-780; Carl Zeiss) using ZEN 2010 software.

### 2.10 Statistics

Values are expressed as mean ± SD. Data were compared by ANOVA. IL-33, TNF-α, IFN-γ, IL-1β mRNA expression data in human myocardial tissue were correlated by using a Spearman correlation (SPSS 18.0, Chicago, IL, USA). Values of p ≤ 0.05 were considered significant.

## 3 Results

### 3.1 IL-33 is constitutively expressed in human cardiac fibroblasts and cardiac myocytes

**Fig. 1:**
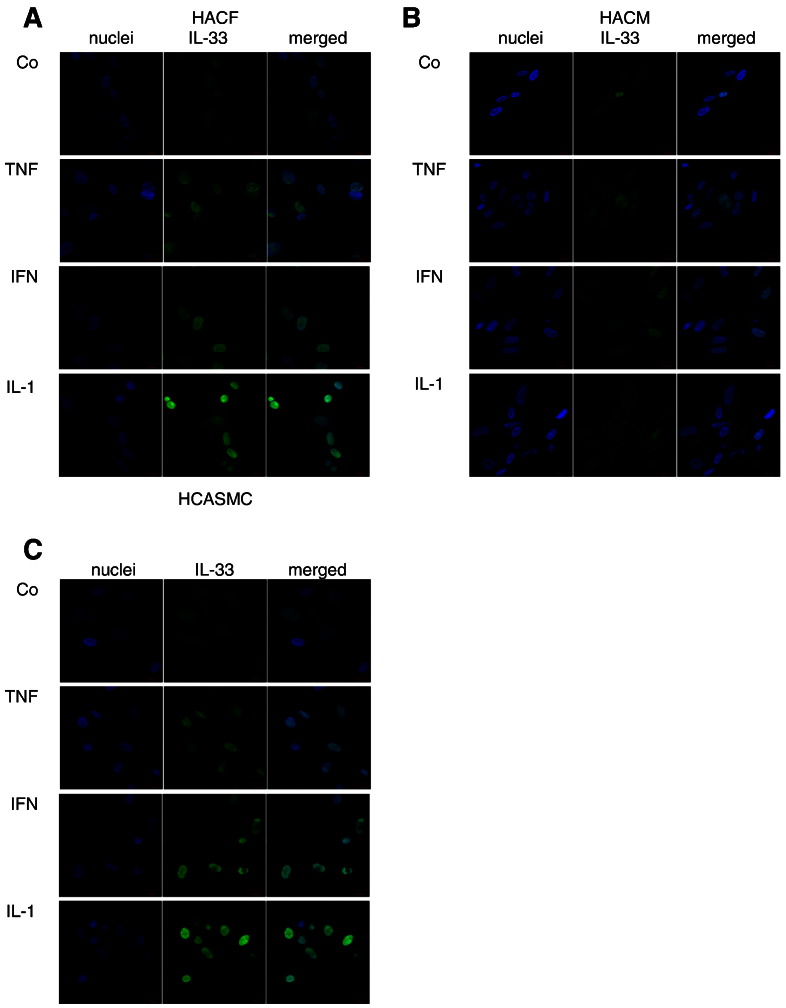
Expression of interleukin (IL)-33 in human cardiac fibroblasts, cardiac myocytes and coronary artery smooth muscle cells. Human adult cardiac fibroblasts (HACF), human adult cardiac myocytes (HACM), and human coronary artery smooth muscle cells (HCASMC) were incubated for 24 hours (h) in the absence (control, Co) or presence of tumor necrosis factor (TNF)-α, interferon (IFN)-γ or IL-1β (each at 2000 Units/mL (U/mL)). Staining for IL-33 in HACF (A), HACM (B) and HCASMC (C) was performed as described in “Materials and methods.” Original magnification × 400. Staining was representative for 2 different donors for each cell types.

**Fig. 2:**
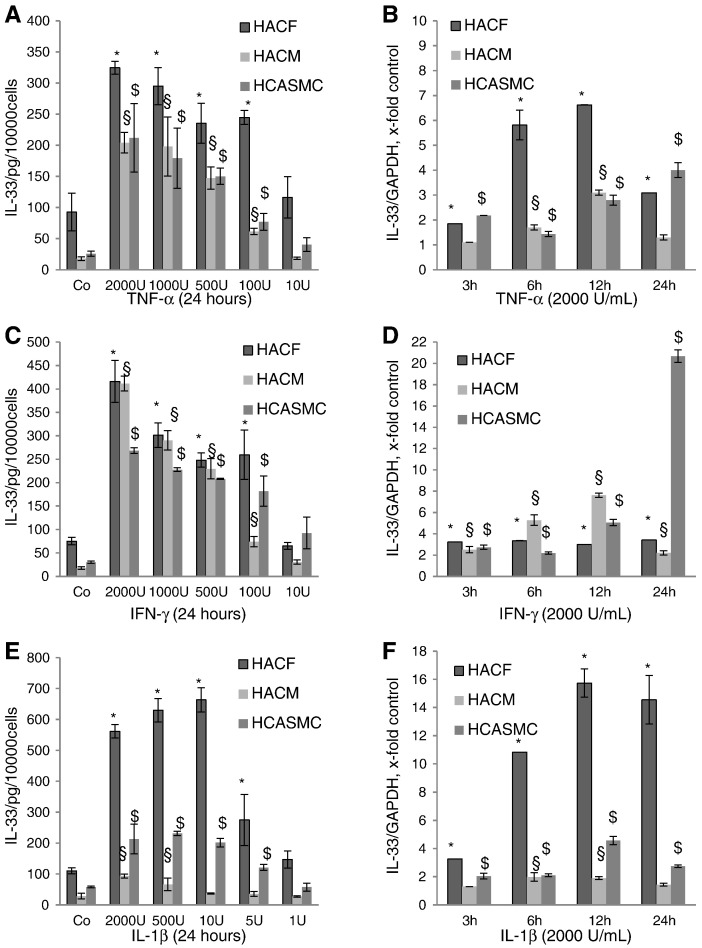
Effects of tumor necrosis factor (TNF)-α, interferon (IFN)-γ and interleukin (IL)-1β on intracellular IL-33 protein expression and IL-33 mRNA in human cardiac fibroblasts, cardiac myocytes and coronary artery smooth muscle cells. HACF, HACM, and HCASMC were incubated for 24 h in the absence or presence of TNF-α (A), IFN-γ (C) or IL-1β (E) at indicated concentrations. IL-33 protein in the cell lysates was measured by a specific ELISA as described in “Materials and methods”. Each experiment was performed in triplicates. Values are given as pg/10,000 cells/24 h and represent mean ± SD. Experiments were performed 4 times with cells obtained from 3 different donors. *p ≤ 0.05 as compared to the control in HACF; §p ≤ 0.05 as compare to the control in HACM; $p ≤ 0.05 as compared to the control in HCASMC. HACF, HACM, and HCASMC were incubated for 3, 6, 12 or 24 h in the absence or presence of TNF-α (B), IFN-γ (D) or IL-1β (F) (each at 2000 Units/mL (U/mL)). mRNA was prepared and RealTime-PCR with primers specific for IL-33 or glyceraldehyde-3-phosphate dehydrogenase (GAPDH) was performed as described in “Materials and methods”. IL-33 mRNA levels were normalized according to the GAPDH mRNA levels. Each experiment was performed in triplicates. Values are given as x-fold of control, which was set as 1, and represent mean ± SD. Experiments were performed 2 times with cells obtained from 2 different donors. *p ≤ 0.05 as compared to the controls in HACF; §p ≤ 0.05 as compared to the controls in HACM; $p ≤ 0.05 as compared to the controls in HCASMC.

As can be seen from Fig. 1, HACF (Fig. 1A) and HACM (Fig. 1B) express IL-33 at protein level as determined by immunofluorescence analysis. IL-33 is expressed in the nucleus of these cells as shown by the co-staining with DAPI (Figs. 1A and B). We also determined IL-33 protein in permeabilized HACF and HACM by ELISA (Figs. 2B, D, F). HCASMC also express IL-33 protein under basal conditions as determined by immunofluorescence analysis (Fig. 1C) and ELISA (Figs. 2B, D, F).

### 3.2 Pro-inflammatory cytokines increase IL-33 expression in human cardiac fibroblasts, cardiac myocytes and vascular smooth muscle cells via NF-κB, MEK or JAK1/2 pathways

We found that TNF-α, IFN-γ, and IL-1β at a concentration of 2000 units/ml (U/mL) increased intracellular IL-33 in HACF (Fig. 1A), HACM (Fig. 1B) and HCASMC (Fig. 1C), as determined by immunofluorescence analysis. When HACF, HACM and HCASMC were incubated for 24 h with TNF-α, IFN-γ or IL-1β at indicated concentrations, we found a concentration-dependent increase in intracellular IL-33 protein in these cells (Figs. 2A, C, E). At the highest tested concentration, TNF-α (Fig. 2A) induced IL-33 protein up to 3.5-, 11.5- and 8.2-fold, IFN-γ (Fig. 2C) up to 5.5-, 23.1- and 8.9-fold, and IL-1β (Fig. 2E) up to 5.1-, 3.3- and 3.7-fold in HACF, HACM and HCASMC, respectively. Stimulatory effects of TNF-α, IFN-γ and IL-1β at 2000 U/mL each on IL-33 protein expression in additional donors are shown in Supplemental Table 2. In all three cell types TNF-α and IFN-γ induced statistically significant (p ≤ 0.05) upregulation of IL-33 protein at concentrations between 2000 and 100 U/mL (Figs. 2A, C). IL-1β statistically significant (p ≤ 0.05) upregulated IL-33 protein at concentration between 2000 and 5 U/mL in HACF and HCASMC (Fig. 2E). In HACM IL-33 was statistically significant (p ≤ 0.05) upregulated after 24 h of incubation with 2000 and 500 U/mL of IL-1β (Fig. 2E). When HACF, HACM and HCASMC were treated with TNF-α, IFN-γ, and IL-1β for 3 h, 6 h, 12 h and 24 h IL-33 mRNA was increased by TNF-α up to 6.6-, 3.1- and 4-fold, by IFN-γ up to 3.4-, 7.6- and 20.7-fold, and by IL-1β up to 15.7-, 2.0- and 4.6-fold in the respective cell type (Figs. 2B, D, F).

**Fig. 3:**
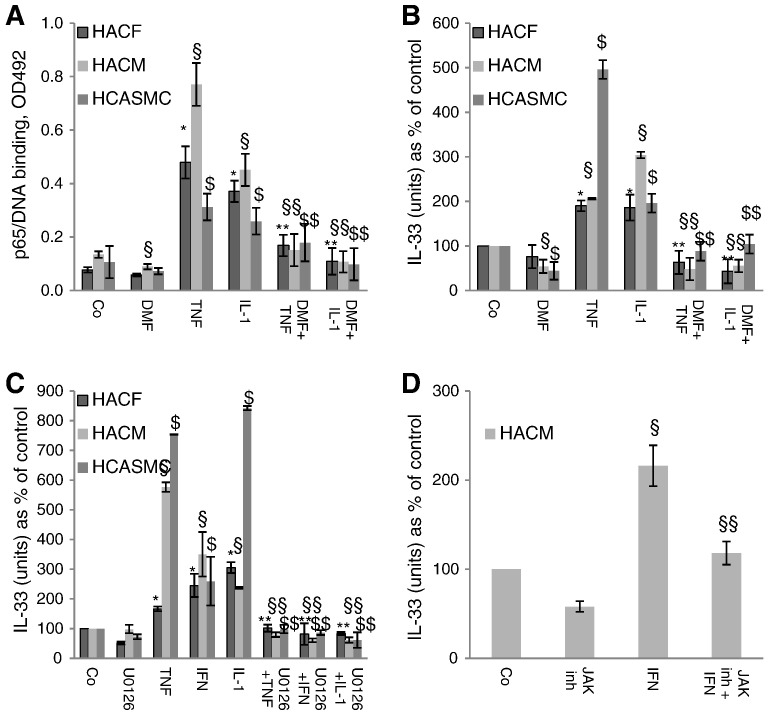
Nuclear factor-κB (NF-κB) inhibitor dimethylfumarate (DMF) abrogated TNF-α- and IL-1β-induced nuclear translocation of NF-κB p65 subunit and IL-33 expression and mitogen-activated protein/extracellular signal-regulated kinase (MEK) or janus-activated kinase (JAK) 1 and 2 inhibitors reduced TNF-α, IFN-γ, or IL-1β-induced IL-33 expression in human adult cardiac fibroblasts (HACF), cardiac myocytes (HACM), or coronary artery smooth muscle cells (HCASMC). HACF, HACM, and HCASMC were pre-incubated for 30 min with DMF at 100 μM. Subsequently, these cells were treated with TNF-α- or IL-1β (each at 2000 U/mL) for 60 min. Preparation of nuclear extracts and quantification of p65 NF-κB subunit (A) were performed as described in “Materials and methods”. Values are given as OD492 nm and represent mean ± SD. Experiments were performed 2 times with cells obtained from 2 different donors for each cell types. HACF, HACM, and HCASMC were first pre-incubated with DMF at 100 μM for 30 min and then treated with TNF-α- or IL-1β for 24 h (B). HACF, HACM, and HCASMC were first pre-incubated with U0126 at 10 μM for 30 min and then treated with TNF-α, IFN-γ or IL-1β for 24 h (C). HACM were first pre-incubated with JAK inhibitor I at 10 μM for 30 min and then treated with IFN-γ at 2000 U/mL for 24 h (D). IL-33 protein in the cell lysates was measured by a specific ELISA as described in “Materials and methods”. Each experiment was performed in triplicates. Values are given in IL-33 (units) as % of control, which was set as 100% and represent mean ± SD. Experiments were performed 3 times with cells obtained from 3 different donors. *p ≤ 0.05 as compared to untreated control in HACF; **p ≤ 0.05 as compared to TNF-α, or IL-1β- or IFN-γ-treated cells in HACF; §p ≤ 0.05 as compare to untreated control in HACM; §§p ≤ 0.05 as compared to TNF-α, or IL-1β- or IFN-γ-treated cells in HACM; $p ≤ 0.05 as compared to untreated control in HCASMC; $$p ≤ 0.05 as compared to TNF-α, or IL-1β- or IFN-γ-treated cells in HCASMC.

The NF-κB inhibitor DMF [42] at 100 μM inhibited TNF-α and IL-1β-induced nuclear translocation of the p65 NF-κB subunit in all three types of the cells (Fig. 3A). Similar results were seen for the NF-κB subunit p50 (data not shown). Furthermore, DMF at the same concentration reduced TNF-α and IL-1β-induced increase in IL-33 protein to control levels in human cardiac fibroblasts, myocytes and vascular smooth muscle cells (Fig. 3B). In contrast, DMF did not influence IFN-γ-induced IL-33 production in any type of the cells used (data not shown). However, MEK inhibitor U0126 at 10 μM (Fig. 3C) or JAK inhibitor I (a JAK 1 and 2 inhibitor) at 10 μM (Fig. 3D) abrogated IFN-γ-induced IL-33 production in these cells. U0126 also abrogated TNF-α and IL-1β-indcued IL-33 expression (Fig. 3C).

### 3.3 Intracellular IL-33 is released during necrosis of human cardiac fibroblasts, myocytes and coronary artery smooth muscle cells

**Fig. 4:**
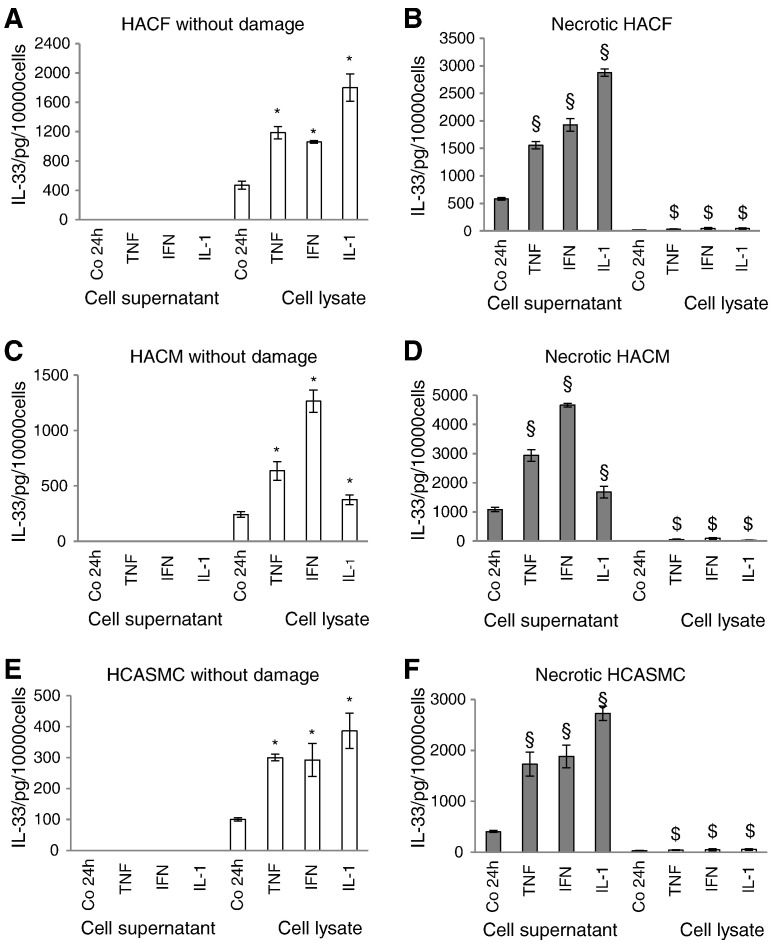
Interleukin (IL)-33 is released during necrosis from human cardiac fibroblasts, cardiac myocytes and coronary artery smooth muscle cells. HACF (panels A and B), HACM (panels C and D), and HCASMC (panels E and F) were incubated for 24 h in the absence or presence of TNF-α, IFN-γ or IL-1β (each at 2000 U/mL). Afterward, cells were left undamaged or necrosis was induced by repeated freeze-thawing. IL-33 protein in the cell culture supernatants and cell lysates was measured by a specific ELISA as described in “Materials and methods”. Each experiment was performed in triplicates. Values are given as pg/10,000 cells/24 h and represent mean ± SD. Experiments were performed 2 times with cells obtained from 2 different donors. Cell culture supernatants — filled bars; cell lysates — open bars. *p ≤ 0.05 as compared to the controls in undamaged cells (cell lysate); §p ≤ 0.05 as compared to the controls in necrotic cells (cell culture supernatants); $p ≤ 0.05 as compared to the controls in necrotic cells (cell lysate).

In culture supernatants of undamaged HACF (Fig. 4A), HACM (Fig. 4C) and HCASMC (Fig. 4E) we could not detect IL-33 protein neither under basal conditions nor after incubation with TNF-α, IFN-γ, or IL-1β. However, when these cells underwent necrosis by repeated freeze-thawing, detectable amounts of IL-33 were released into cell culture supernatants in these cells which were significantly higher when the cells had been treated prior to induction of necrosis with TNF-α, IFN-γ, or IL-1β as compared to the respective controls (Figs. 4B, D, and F). In parallel, in necrotic HACF, HACM and HCASMC intracellular IL-33 concentrations were reduced (Figs. 4B, D, and F).

### 3.4 Expression of IL-33 mRNA in human myocardial tissue correlates with mRNA for pro-inflammatory cytokines

**Fig. 5:**
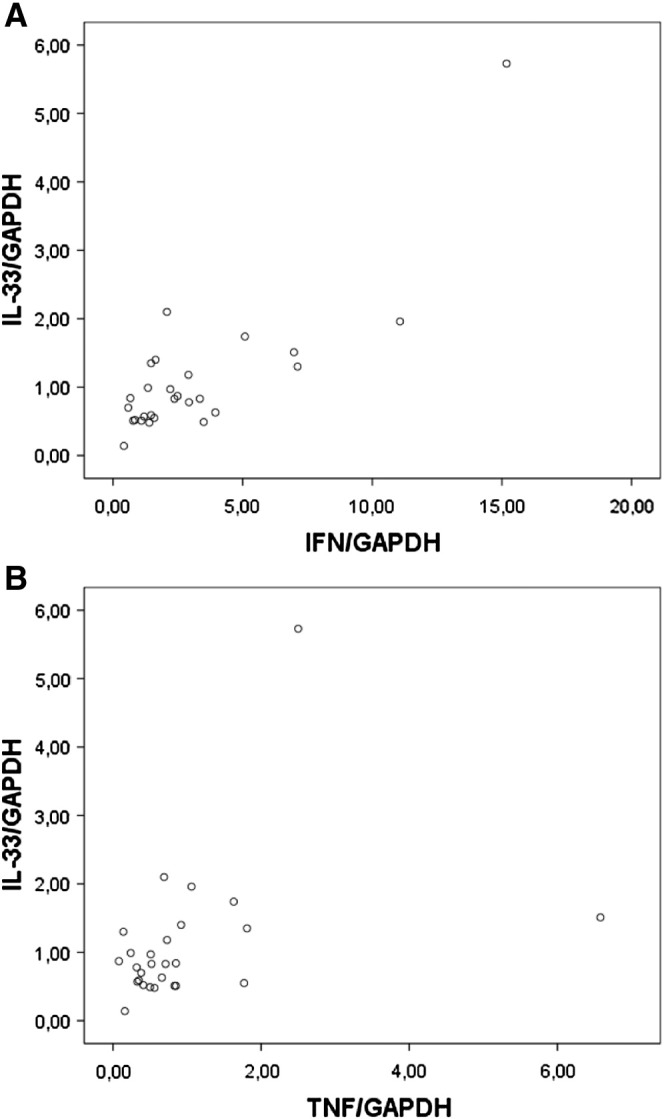
IL-33 mRNA correlates with IFN-γ and TNF-α mRNA expression in human myocardial tissue. RNA was isolated from tissue samples obtained from left ventricles from explanted hearts of patients (n = 27) undergoing heart transplantation. IL-33, IFN-γ and TNF-α mRNA and mRNA for GAPDH was determined by RealTime-PCR as described in “Materials and methods”. mRNA levels of IL-33, IFN-γ and TNF-α were correlated after adjustment for GAPDH.

To test if our *in vitro* results are also relevant for the *in vivo* situation, we measured IL-33, TNF-α, IFN-γ and IL-1β mRNA expression in tissue samples obtained from left ventricles from explanted hearts of patients (n = 27) undergoing heart transplantation. We detected IL-33 mRNA in all samples and found that IL-33 mRNA positively and significantly correlated with IFN-γ (r = 0.591, p = 0.001; Fig. 5A) and TNF-α (r = 0.408, p = 0.035; Fig. 5B) mRNA levels, respectively. We also found a weak positive correlation (r = 0.344) between IL-33 and IL-1β mRNA expression. However, this correlation did not reach statistical significance (p = 0.092; data not shown).

### 3.5 Endothelial cells but not cardiac myocytes, cardiac fibroblasts and smooth muscle cells predominantly express the IL-33 receptor ST2 and secrete sST2

**Fig. 6:**
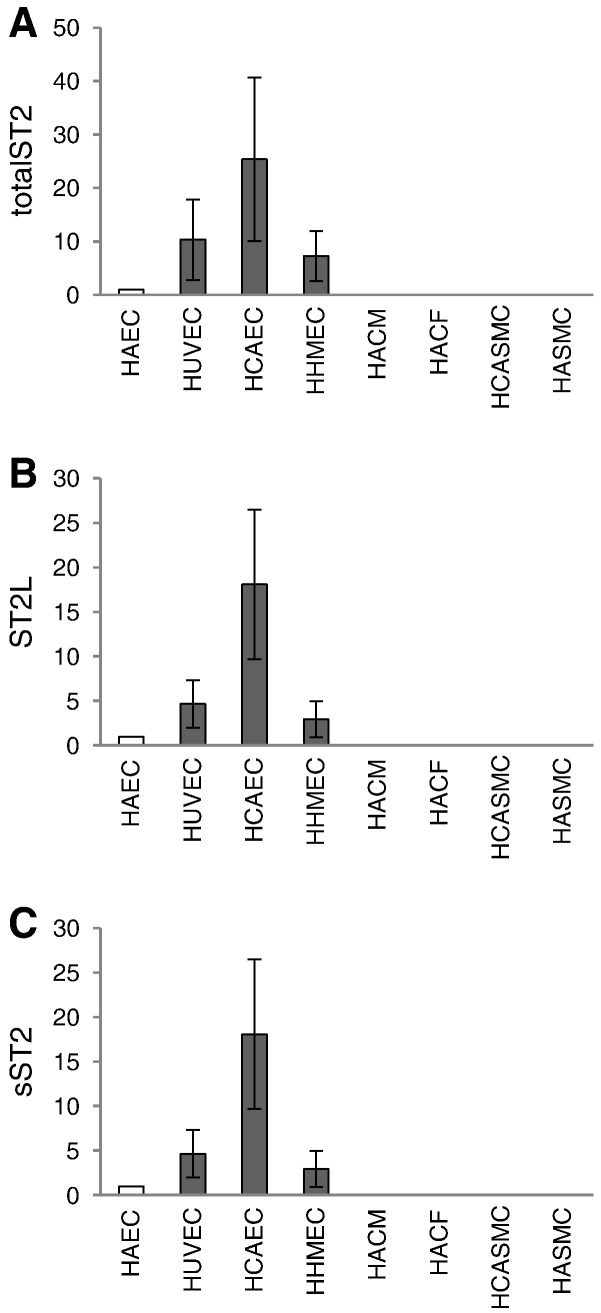
Expression of ST2 receptor isoforms in human cardiac cells and in vascular cells. Human adult cardiac fibroblasts (HACF), human adult cardiac myocytes (HACM), human aortic smooth muscle cells (HASMC), human coronary artery smooth muscle cells (HCASMC), human umbilical vein endothelial cells (HUVEC), human coronary artery endothelial cells (HCAEC), human aortic endothelial cells (HAEC), and human heart microvascular endothelial cells (HHMEC) were left untreated. mRNA was prepared, cDNA was additionally eluted with MinElute PCR Purification Kit and equal amount of cDNA was used for RealTime-PCR with primers specific for totalST2 (A), ST2L (B), or sST2 (C) as described in “Materials and methods”. Values are given as x-fold of HAEC, which was set as 1 and represent mean ± SD. Experiments were performed with cells obtained from at least 3 different donors for each type of cells.

In order to determine which cell types are a possible target for IL-33 or a source of sST2 in the human cardiovascular system we screened different cell types such as cardiac myocytes and fibroblasts and vascular smooth muscle cells and endothelial cells from various vascular beds for the expression of total ST2 mRNA, ST2L mRNA and sST2 mRNA as well as for the secretion of sST2 antigen. As can be seen from Fig. 6, HACF, HACM, HASMC or HCASMC, respectively, express only minor amounts of specific mRNA for total ST2 (Fig. 6A), ST2L (Fig. 6B) or sST2 (Fig. 6C). In comparison to these cell types macrovascular (HUVEC, HCAEC, HAEC) and microvascular (HHMEC) endothelial cells express high levels of mRNA specific for the respective receptor isoforms (Figs. 6A–C). Furthermore, using the ELISA described under “Materials and methods” (lower limit of detection: 31 pg/ml), sST2 protein could not be detected in the supernatants of HACF, HACM, HASMC or HCASMC (Table 1). In contrast, macrovascular (HUVEC, HCAEC, HAEC) and microvascular (HHMEC) endothelial cells secreted sST2 antigen into the respective cell culture supernatant (Table 1).

**Table 1:** Secretion of sST2 by human cardiac and vascular cells.

Cell type	sST2, pg/10,000 cells/24 h
HUVEC	1308 ± 86
HAEC	119 ± 174
HCAEC	219 ± 113
HHMEC	28 ± 10
HACF	n.d.
HACM	n.d.
HCASMC	n.d.
HASMC	n.d.

Confluent monolayers of human umbilical vein endothelial cells (HUVEC), human aortic endothelial cells (HAEC), human coronary artery endothelial cells (HCAEC), human heart microvascular endothelial cells (HHMEC), human adult cardiac fibroblasts (HACF), human adult cardiac myocytes (HACM), human coronary artery smooth muscle cells (HCASMC), and human aortic smooth muscle cells (HASMC) were cultivated under respective culture conditions for 24 h untreated. Cell culture supernatants were collected and sST2 protein was measured using specific ELISA as described in “Materials and methods”. Values are given as pg/10,000 cells and represent mean ± SD in case of HUVEC, HCAEC and HAEC. Experiments were performed with cells obtained from 4 different donors for HUVEC, HACF, HACM, HCASMC, and obtained from 3 different donors for HASMC, HCAEC, HAEC, and HHMEC. N.d. — not detectable.

### 3.6 In human cardiac myocytes, cardiac fibroblasts and vascular smooth muscle cells, IL-33 has neither an effect on NF-κB nuclear translocation nor on IL-6, IL-8 and MCP-1 production

Neither HACF, nor HACM nor HCASMC responded to IL-33 (Supplemental Fig. 1). IL-33 at 100 ng/mL did not induce nuclear translocation of NF-κB p50 (Supplemental Fig. 1A) and p65 (Supplemental Fig. 1B) subunits between 15 min and 60 min of incubation in these cells. When HACF, HACM or HCASMC were incubated with IL-33 at concentrations of 1, 10 or 100 ng/mL for 24 h, no changes in IL-6 (Supplemental Fig. 1C), IL-8 (Supplemental Fig. 1D) or MCP-1 (Supplemental Fig. 1E) protein production or mRNA expression (data not shown) were observed. In accordance with recently published results from our group in the same set of experiments, rh IL-33 used at the same concentrations significantly upregulated IL-6, IL-8 and MCP-1 as well as NF-κB p50 and p65 nuclear translocation in HUVEC and HCAEC [11]. In contrast to IL-33, IL-1β at 10 ng/mL increased the production of IL-6 (Supplemental Fig. 2A), IL-8 (Supplemental Fig. 2B) and MCP-1 (Supplemental Fig. 2C) in HACF, HACM and HCASMC ().

### 3.7 Cellular localization of IL-33 and ST2 protein in human heart

**Fig. 7:**
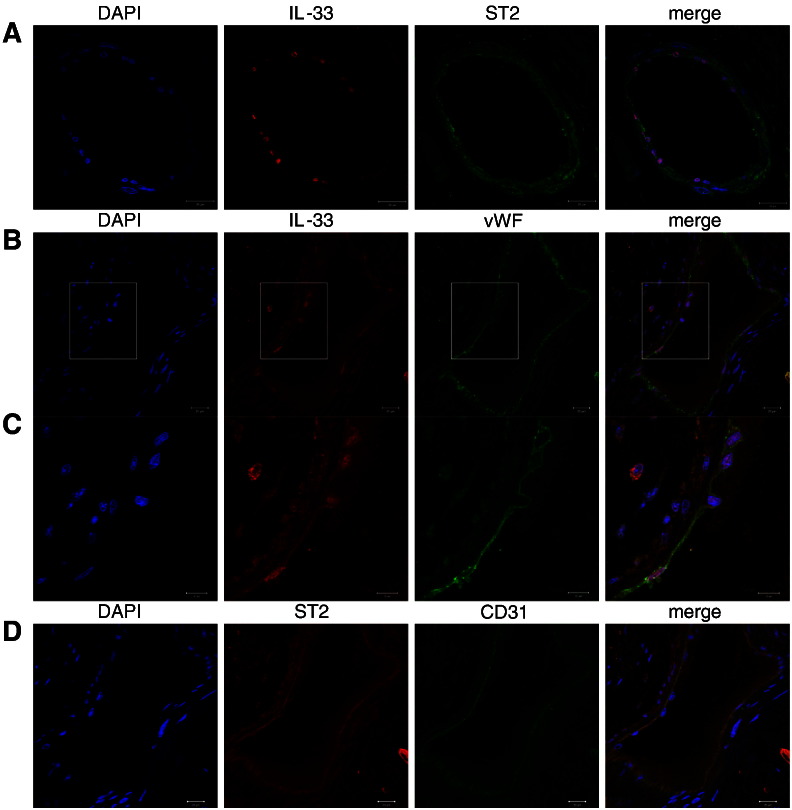
IL-33 and ST2 protein in endothelial cells in human heart. Confocal immunofluorescence images of blood vessels in human heart tissue. Co-staining of IL-33 and ST2 (A), IL-33 and von Willebrand factor (vWF, B and C), and ST2 and CD31 (D). Original magnification × 630. C, cropped region of B with factor 1.5. Scale bar = 20 μm (A, B, D). Scale bar = 10 μm (C).

As can be seen from Fig. 7A, nuclear IL-33 and membrane bound ST2 are expressed on the same cells in blood vessels in the heart. Co-staining with vWF or CD31 as specific markers of endothelial cells showed that both IL-33 (Figs. 7B and C) and ST2 (Fig. 7D) are strongly expressed by endothelial cells in the vessel wall. IL-33 protein is also expressed by cardiac myocytes (Supplemental Fig. 3A, 3C, and Supplemental Fig. 5) and smooth muscle cells (Supplemental Fig. 4). Supplemental Fig. 3A shows that also white blood cells are positive for IL-33 protein (indicated with the arrow) as already described previously [1]. However, ST2 protein expression is absent in SMA-positive cells (Supplemental Fig. 4A) and troponin-positive cells are only weakly positive for ST2 protein (Supplemental Fig. 3B and 3C) in human heart confirming our *in vitro* findings (Fig. 1, Fig. 6).

## 4 Discussion

IL-33 was found recently in endomyocardial biopsies from patients with aortic valve stenosis and congestive heart failure [38]. In addition IL-33 is expressed by endothelial and smooth muscle cells [1], [8], [9], [11], [14], [15], [31], [38]. We show here for the first time that human adult cardiac fibroblasts and myocytes constitutively express IL-33 *in vitro*. In our study IL-33 protein was localized in the nucleus but could not be detected in the supernatants of cultured undamaged cardiac myocytes and fibroblasts. Moreover, we show that the proinflammatory cytokines TNF-α, IFN-γ and IL-1β upregulate IL-33 on the protein as well as on the mRNA level in cardiac myocytes, fibroblasts and vascular smooth muscle cells. The concentrations of TNF-α, IFN-γ and IL-1β inducing an increase in IL-33 production in those cells *in vitro* were higher than plasma concentrations of these cytokines measured in patients suffering from various cardiovascular diseases such as heart failure, myocardial infarction, or unstable angina [44], [45], [46]. It should be taken into consideration, however, that in an *in vivo* setting these cytokines might accumulate at sites of inflammation or tissue damage leading to higher local concentrations of these biomolecules in the affected tissue as compared to systemic levels in the plasma. In that respect it is of interest that higher levels of TNF-α were found in hearts from patients suffering from end stage heart failure as compared to plasma levels of TNF-α seen in such patients. In addition, the respective levels of the cytokine were dramatically increased when compared to levels in healthy controls [47]. Furthermore it should be noted, that the concentrations of TNF-α, IFN-γ and IL-1β used here in our cell culture experiments are in the same range as concentrations of these cytokines used in other cell culture studies performed by us and others [39], [40], [48] In our *in vitro* experiments, similar to other proteins investigated in earlier studies, we noted a donor-dependent variability of basal and cytokine-induced IL-33 expression in the cells tested which might reflect variability in the biosynthesis of IL-33 amongst different individuals. In that respect it is of interest that IL-33 plasma levels varied from 5.4 to 17893.0 pg/mL in patients with myocardial infarction [49]. It should be emphasized, however, that all cells responded with a significant increase in IL-33 in response to TNF-α, IFN-γ and IL-1β. In agreement with our results IL-33 expression was previously shown to be induced by proinflammatory stimuli in various cell types such as dermal, lung or synovial fibroblasts and human airway smooth muscle cells [1], [15], [50] as well as keratinocytes, stellate cells, intestinal epithelial cells, astrocytes, and adipocytes [1], [15], [18], [19], [34], [35]. In human umbilical artery smooth muscle cells IL-33 was induced by IL-1 and IFN-γ, but not by TNF-α [36]. In contrast to our findings, however, TNF-α and IL-1β did not induce IL-33 expression in rat neonatal cardiac myocytes and fibroblasts [16]. This could reflect a species specific and/or development-dependent regulation of IL-33.

As shown by immunohistochemical analysis of hearts of the patients suffering from cardiomyopathy endothelial cells in the heart express nuclear IL-33 as well as its membrane-bound receptor ST2, whereas cardiac myocytes and vascular smooth muscle cells in the heart stained positive for IL-33 only and were weak positive or negative, respectively, for ST2 confirming our *in vitro* observations. In explanted hearts of patients undergoing heart transplantation IL-33 mRNA levels correlated positively with the levels of mRNA specific for the inflammatory mediators TNF-α and IFN-γ, respectively. It is of interest that previously a positive correlation between IL-33 and TNF-α expression in the lung tissues in patients with asthma has been described suggesting a role of IL-33 in the pathogenesis of that disease [15].

Numerous studies have investigated pathways activated by IL-33 in different cells [26]. In contrast, little is known about the regulation of IL-33 expression by various cytokines. Different signaling pathways are activated by TNF-α, IL-1β or IFN-γ [51], [52]. In our study, the effects of TNF-α and IL-1β on IL-33 expression were mediated via NF-κB and MEK whereas the increase in IL-33 expression by IFN-γ was mediated through MEK and JAK 1 and 2 pathways. In agreement with our results it was shown, that the induction of IL-33 expression by IL-1 in human pancreatic stellate cells was also mediated via NF-κB [34]. Furthermore, similar to our results, IFN-γ-induced expression of IL-33 in human epidermal keratinocytes was found to be MEK- and JAK 1- and 2-dependent [53].

It is still a matter of controversy if and how IL-33 is secreted by undamaged cells. We could not detect any secreted IL-33 protein in the supernatant of undamaged unstimulated human cardiac fibroblasts and cardiac myocytes. Recently, it was shown that PMA induced the secretion of IL-33 in rat neonatal cardiac fibroblasts [16]. As described above, TNF-α, IFN-γ or/and IL-1β were shown to induce intracellular accumulation of IL-33, but not its extracellular secretion, from intestinal epithelial cells [18], synovial fibroblasts [50], pancreatic stellate cells [34] and adipocytes/preadipocytes [35]. These observations are consistent with ours in human cardiac fibroblasts and myocytes, as we did not detect IL-33 protein in the supernatant of undamaged TNF-α-, IFN-γ-, or IL-1β-treated cells. Nuclear IL-33 expression was previously found in endothelial and epithelial cells in normal, chronically inflamed, and tumor tissues [8], [9], [11], [14], [18], pancreatic stellate cells [34], keratinocytes [54], and astrocytes [19]. Dermal and pulmonary fibroblasts showed nuclear IL-33 expression in patients with systemic sclerosis [54]. In human monocytes the IL-33 protein was mainly found in the cytoplasm [37]. In contrast, different murine and human primary cells or cell lines secrete IL-33 after stimulation with LPS, PMA plus ionomycin, Toll-like receptor (TLR)7 or TLR1/2 agonists, IgE, or infection with influenza A virus [19], [20], [21], [22], [55]. Activated murine macrophages and dendritic cells release IL-33 protein following cellular necrosis [24], [37] and noradrenaline and adrenaline enhanced IL-33 production by necrotic dendritic cells upon LPS stimulation [56]. Therefore, IL-33 is recognized as a dual function cytokine that acts either intracellular to regulate gene transcription or extracellular via binding to its receptor ST2L [9], [57].

Upon cell injury, endogenous danger signals, so-called DAMPs, are released by necrotic cells [25]. IL-33 is recognized as a key danger signal released by necrotic structural cells [23], [24], [41], [57]. When human cardiac cells as well as coronary artery smooth muscle cells were subjected to necrosis in our study, IL-33 was released into cell culture supernatants. Previously, IL-33 was shown to be released from necrotic cells of stromal origin such as mouse embryonal fibroblasts and smooth muscle cells [41]. IL-33 is also released extracellular after endothelial cell damage or injury [23]. Upon release, IL-33 was shown to be recognized by different immune cells such as mast cells, basophils, eosinophils, nuocytes and Th2 cells, which produce proinflammatory mediators in response to IL-33 [1], [26], [27], [41]. Extracellular IL-33 can also induce endothelial activation by upregulation of adhesion molecules ICAM-1, VCAM-1, E-selectin and induction of adhesion of blood leukocytes to activated endothelial cell monolayers [11]. Thus, it is possible to speculate that IL-33 could be released during necrosis or damage from human adult cardiac cells and drive the immune answer of haematopoietic cells or modulate functions of neighboring cells in a paracrine manner. It should also be noted that recently it was shown that IL-33 is released from fibroblasts following mechanical strain [58]. If such a mechanism is also operative in cardiac cells, one might speculate that IL-33 might also play a pathophysiological role in cardiovascular diseases such as heart failure characterized by mechanical strain impacting on the cardiac tissue.

In order to identify possible cellular targets for IL-33 produced by cardiac myocytes and fibroblasts in the cardiovascular system we screened different cell types isolated from human heart and adjacent vascular beds for the presence of specific mRNA for ST2 isoforms and for the production of sST2 protein. We show that macrovascular endothelial cells isolated from aorta and coronary arteries and microvascular endothelial cells isolated from myocardial tissue express total ST2, ST2L and sST2 mRNA and secrete sST2. In contrast, cardiac myocytes and cardiac fibroblasts or vascular smooth muscle cells from coronary arteries or aorta express only minor levels of total ST2, ST2L and sST2 mRNA and do not secrete detectable amount of sST2 antigen. In agreement with our results sST2 mRNA and protein and ST2L mRNA was previously demonstrated by others in both vein and arterial endothelial cells as well as in microvascular endothelial cells from lung blood vessels in humans [30], [31], [32], [38], [59]. A study by Bartuneck et al. found no transmyocardial sST2 gradient, which argues against cardiac production of serum sST2 [38]. Similar to our findings Mildner et al. detected neither sST2 mRNA expression nor protein production in human cardiac fibroblasts. However, in the same paper the group showed sST2 mRNA expression and sST2 protein production in human cardiac myocytes [32]. These latter differences could reflect differences in cell culture conditions. Both sST2 and ST2L mRNA were detected in neonatal rat cardiac cells [16], [60], which could indicate a species-specific and/or development-depending cellular distribution of ST2.

Upon interaction with its receptor IL-33 was shown to activate NF-κB in different cell types [1], [11]. Moreover, IL-33 induces inflammatory activation, as shown by increased IL-6, IL-8 or MCP-1 production, in cell types which express ST2L such as endothelial cells, epithelial cells, mast cells, basophils and eosinophils [11], [26], [30]. Consistent with the scarce expression of ST2L in human cardiac myocytes and fibroblasts and in vascular smooth muscle cells in our study, neither NF-κB nuclear translocation nor IL-6, IL-8 or MCP-1 production was affected in such cells treated with rh IL-33 in concentrations previously shown to be effective in *in vitro* studies by us and others [11], [30], [33]. Recently, rat adult cardiac fibroblasts were shown to express ST2 and responded to treatment with IL-33 with increased IL-6 and MCP-1 production and activation of the NF-κB p65 subunit [61], which again supports the notion of species-specific differences in ST2 expression and in the cellular response to IL-33.

In conclusion, we found that IL-33 is expressed by human adult cardiac myocytes and fibroblasts and by human coronary artery smooth muscle cells and that the cytokine is present in the nucleus of these cells. The IL-33 receptor ST2 shows a distinct expression pattern in the heart as it is predominantly expressed by endothelial cells of the cardiac vasculature. IL-33 is upregulated by TNF-α, IFN-γ and IL-1β and is released during necrosis of human cardiac and smooth muscle cells. In human myocardial tissue from hearts of patients undergoing heart transplantation, endothelial cells are the main cell type expressing both IL-33 as well as its receptor ST2. Furthermore IL-33 expression correlates positively with that TNF-α and IFN-γ, respectively, in such myocardial tissue. Possible pathophysiological consequences of the regulated expression of the alarmin IL-33 and the cell specific distribution of its receptor ST2 in the human heart e.g. following injury and subsequent necrosis warrant subsequent investigations to further test our hypothesis in the *in vivo* setting.

## Disclosure statement

None declared.
