# Supplementary material for: Components of the interleukin-33/ST2 system are differentially expressed and regulated in human cardiac cells and in cells of the cardiac vasculature
Source: J Mol Cell Cardiol. 2013 Jul;60:16–26. doi: 10.1016/j.yjmcc.2013.03.020 (PMC3683148; doi:10.1016/j.yjmcc.2013.03.020)
Supplement: Supplementary file 1 — Supplementary material [file mmc1.doc]

**Supplemental material**

**Demyanets et al. „Components of the interleukin-33/ST2 system are differentially expressed and regulated in human cardiac cells and in cells of the cardiac vasculature.”**

Supplemental Table 1.Primers used.

| Gene | forward primer | reverse primer | UPLprobe | Amplicon Size [bp] |
| --- | --- | --- | --- | --- |
| GAPDH | agccacatcgctcagacac | gcccaatacgaccaaatcc | #60 | 66 |
| Total ST2 | ttgtcctaccattgacctctacaa | gatccttgaagagcctgacaa | #56 | 75 |
| ST2L | gaaatacctgagactgggtgatttat | gaagtgcctgcctttgctt | #29 | 149 |
| sST2 | gggagagatatgctacctggag | cgcctgctctttcgtatgtt | #86 | 68 |
| IL-33 | agcaaagtggaagaacacagc | cttctttggccttctgttgg | #33 | 74 |
| TNF-α | cagcctcttctccttcctgat | gccagagggctgattagaga | #29 | 123 |
| IFN-γ | ggcattttgaagaattggaaag | tttggatgctctggtcatctt | #21 | 112 |
| IL-1β | tacctgtcctgcgtgttgaa | tctttgggtaatttttgggatct | #78 | 76 |
| MCP-1 | ttctgtgcctgctgctcat | ggggcattgattgcatct | #83 | 73 |
| IL-6 | gatgagtacaaaagtcctgatcca | ctgcagccactggttctgt | #40 | 130 |
| IL-8 | agacagcagagcacacaagc | atggttccttccggtggt | #72 | 62 |

*Supplemental Table 2. Stimulatory effects of IL-1β, TNF-α or IFN-γ on IL-33 protein production in human cardiac and smooth muscle cells derived from different donors*

| Donors | HACF | | | HACM | | | HCASMC | | |
| --- | --- | --- | --- | --- | --- | --- | --- | --- | --- |
|  | IL-1β | TNF-α | IFN-γ | IL-1β | TNF-α | IFN-γ | IL-1β | TNF-α | IFN-γ |
| Nr. 1 | 1.9 | 2.1 | 4.6 | 1.4 | 1.9 | 2.5 | 1.4 | 2.6 | 6.3 |
| Nr. 2 | 1.5 | 2.6 | 3.3 | 2.4 | 5.8 | 8.7 | n.d. | n.d. | n.d. |
| Nr. 3 | n.d. | n.d. | n.d. | 1.5 | 1.7 | 3.0 | n.d. | n.d. | n.d. |
| Nr. 4 | n.d. | n.d. | n.d. | 1.4 | 2.7 | 3.6 | n.d. | n.d. | n.d. |
| Nr. 5 | n.d. | n.d. | n.d. | 1.6 | 2.6 | 5.2 | n.d. | n.d. | n.d. |
| Nr. 6 | 3.8 | 2.5 | 2.3 | n.d. | n.d. | n.d. | n.d. | n.d. | n.d. |
| Nr. 7 | n.d. | n.d. | n.d. | n.d. | n.d. | n.d. | 1.3 | 2.0 | 6.3 |
| Nr. 8 | n.d. | n.d. | n.d. | n.d. | n.d. | n.d. | 8.4 | 7.5 | 2.6 |
| Nr. 9 | n.d. | n.d. | n.d. | n.d. | n.d. | n.d. | 3.9 | 3.0 | 3.0 |

Human adult cardiac fibroblasts (HACF), human adult cardiac myocytes (HACM), and human coronary artery smooth muscle cells (HCASMC) were incubated for 24 hours in the absence or presence of IL-1β, TNF-α, or IFN-γ at 2000 Units (U)/mL each. IL-33 protein in the cell lysates was measured by a specific ELISA as described in “Materials and Methods”. Each experiment was performed in triplicates. Values are given as x-fold of respective controls. n.d. – not determined.

| **A** | 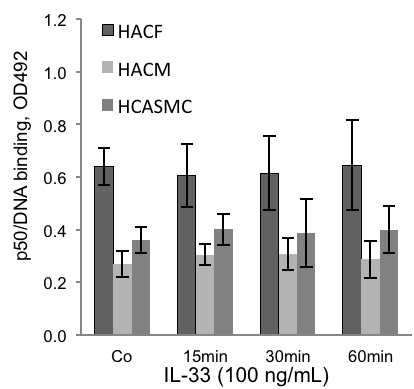 | **B** | 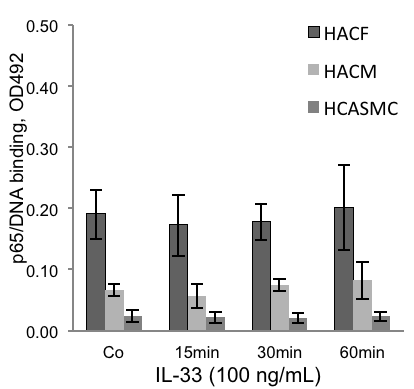 |
| --- | --- | --- | --- |
| **C** | 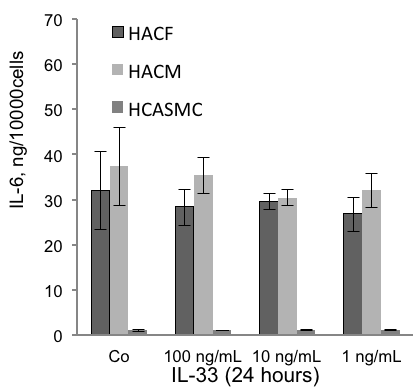 | **D** | 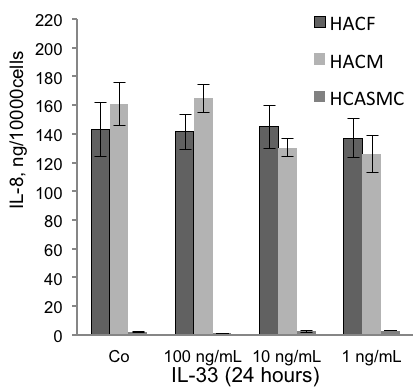 |
| **E** | 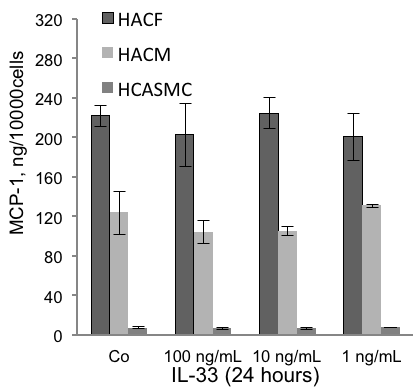 |  |  |

**Supplemental Figure 1.***Interleukin (IL)-33 does neither modulate nuclear factor-κB (NF-κB) p50 and p65 subunit nor IL-6, IL-8 and monocyte chemoattractant protein (MCP)-1 levels in human adult cardiac fibroblasts (HACF), cardiac myocytes (HACM), or coronary artery smooth muscle cells (HCASMC).*

HACF, HACM, and HCASMC were incubated for 15, 30, or 60 min in the absence or presence of IL-33 at 100 ng/mL. Preparation of nuclear extracts and quantification of p50 NF-κB subunit (A) and p65 NF-κB subunit (B) were performed as described in “Materials and Methods”. Values are given as OD492 nm and represent mean ± SD. Experiments were performed 2 times with cells obtained from 3 different donors for each cell types. HACF, HACM, and HCASMC were incubated for 24 h in the absence or presence of IL-33 at 1, 10 and 100 ng/mL. IL-6 (C), IL-8 (D) and MCP-1 (E) antigens were measured in cell culture supernatants using specific ELISAs as described in “Materials and Methods”. Each experiment was performed in triplicates. Values are given as ng/10000 cells/24 h and represent mean ± SD. Experiments were performed 3 times with cells obtained from 3 different donors.

| **A** | 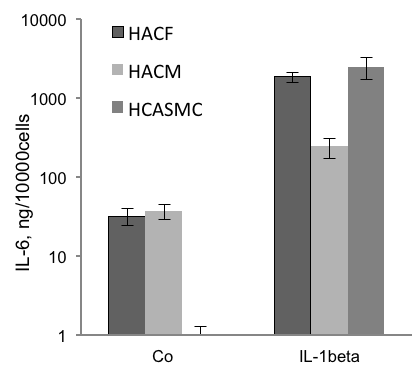  *  §  $ | **B** | 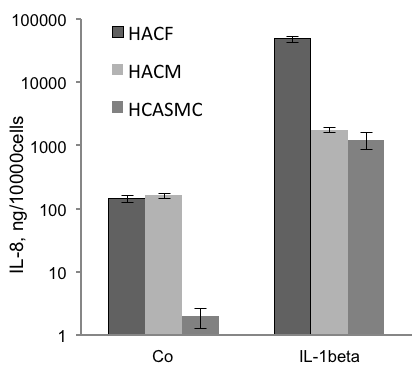  *  §  $ |
| --- | --- | --- | --- |
| **C** | 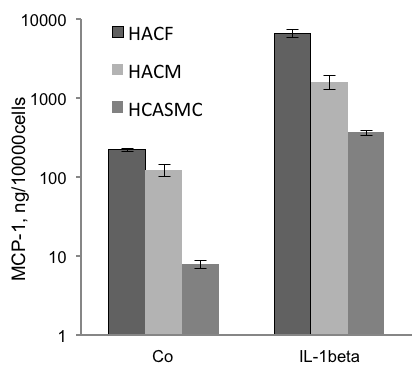  *  §  $ |  |  |

**Supplemental Figure 2.***IL-1β-induced IL-6, IL-8 and MCP-1 production in human adult cardiac fibroblasts (HACF), human adult cardiac myocytes (HACM) and human coronary artery smooth muscle cells (HCASMC).*

HACF, HACM, and HCASMC were incubated for 24 hours (h) in the absence or presence of IL-1β at 10 ng/mL. IL-6, IL-8 and MCP-1 antigens were measured in cell culture supernatants using specific ELISAs as described in “Materials and Methods”. Each experiment was performed in triplicates. Values are given as ng/10000 cells/24 h and represent mean ± SD. Experiments were performed 3 times with cells obtained from 3 different donors.*p≤0.05 as compared to the controls in HACF; §p≤0.05 as compared to the controls in HACM; $p≤0.05 as compared to the controls in HCASMC. Note: The Y-axis is presented in logarithmic scale.

|  | DAPI | IL-33 | merge |
| --- | --- | --- | --- |
| A | 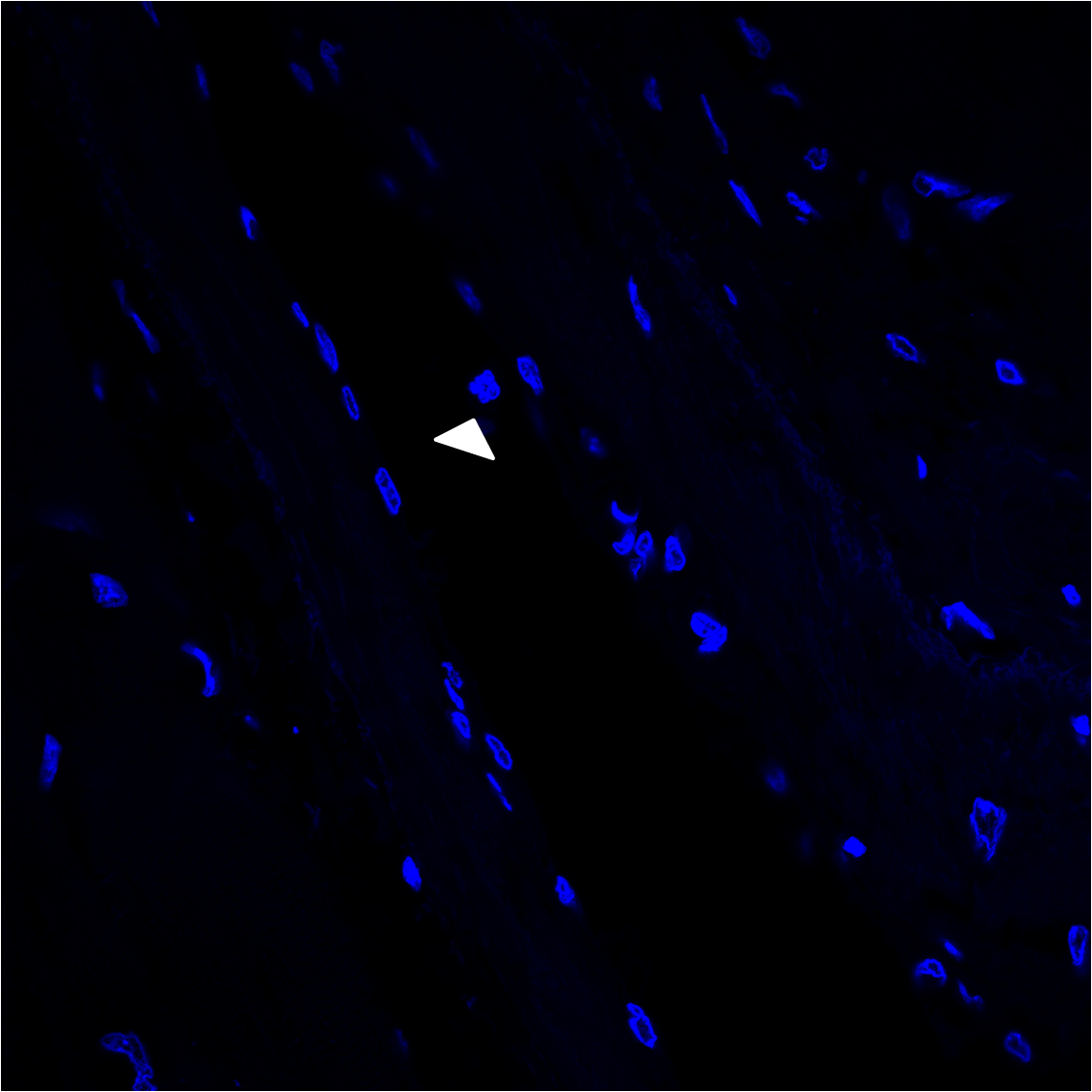 | 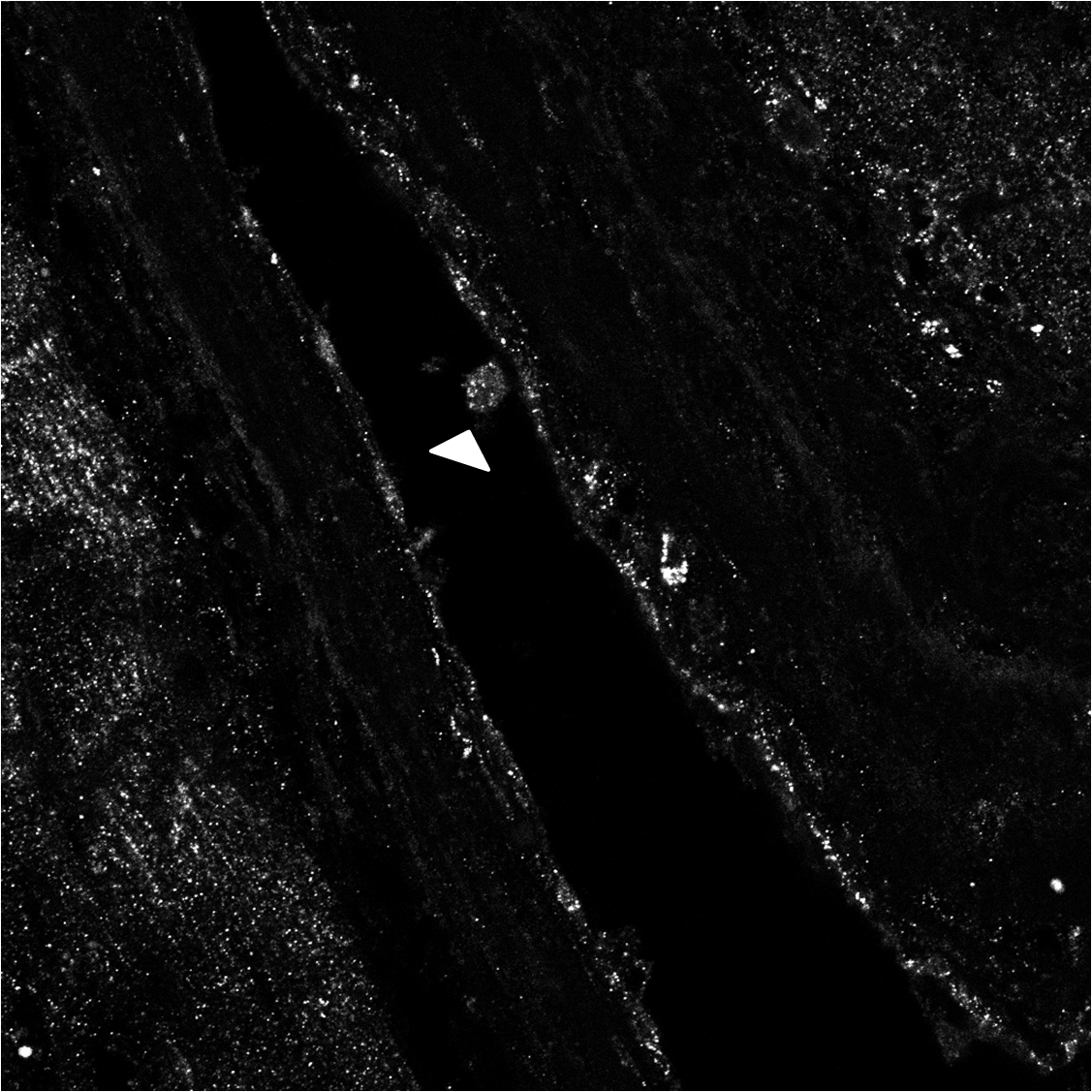 | 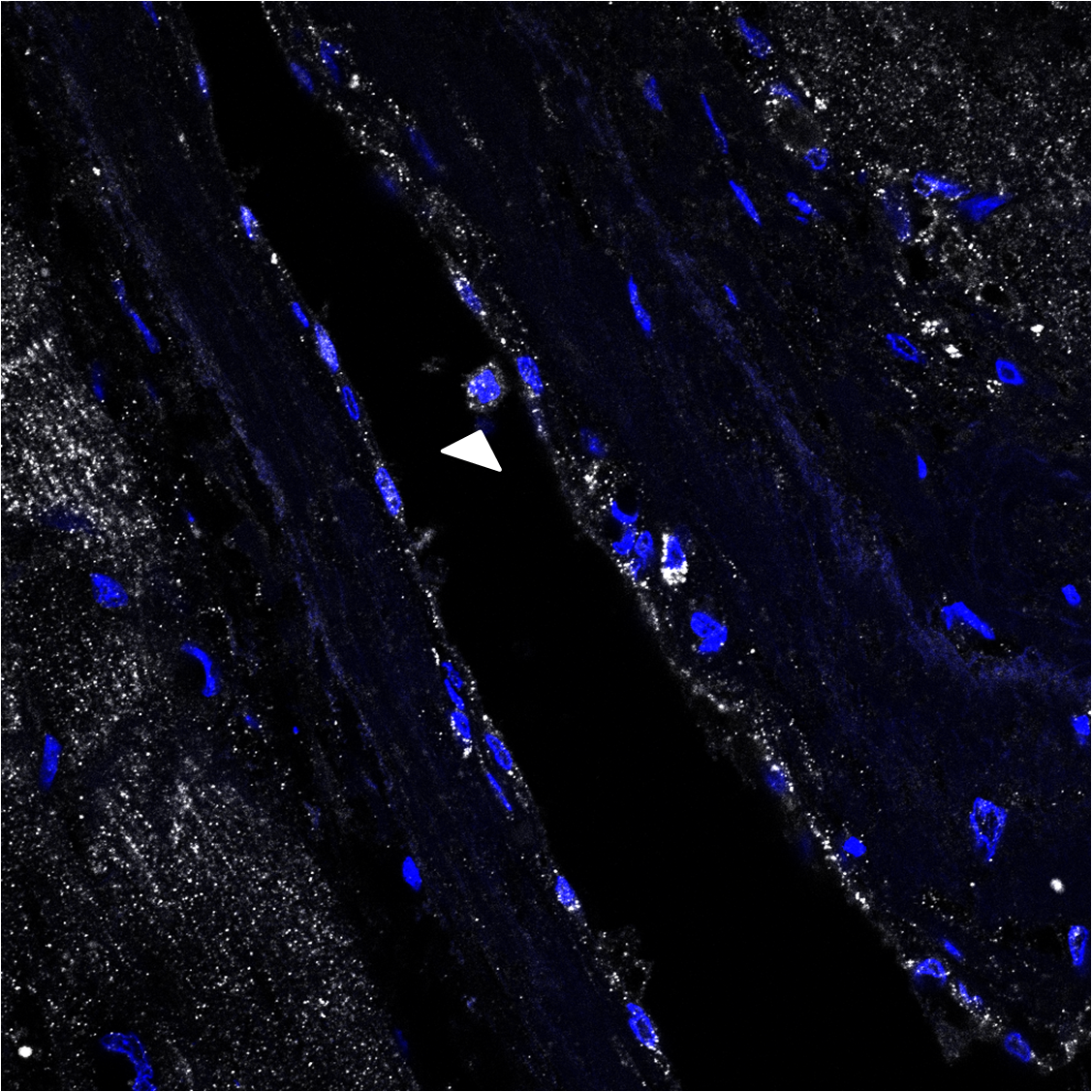 |
|  | DAPI | ST2 | merge |
| B | 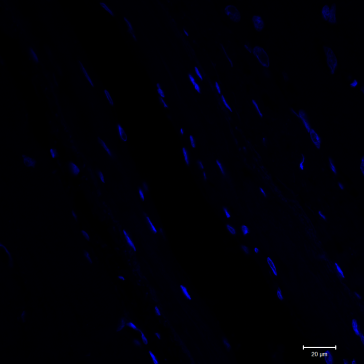 | 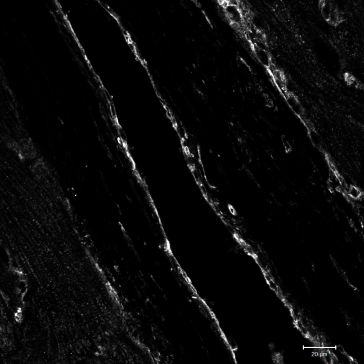 | 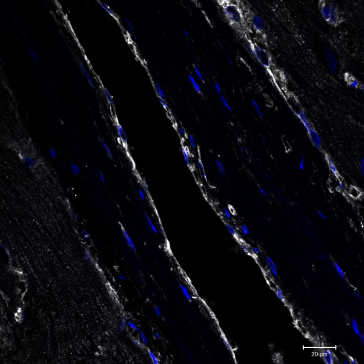 |
|  | DAPI | Troponin | merge |
| C | 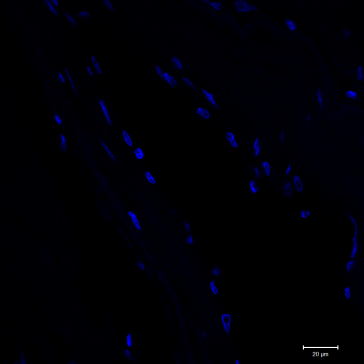 | 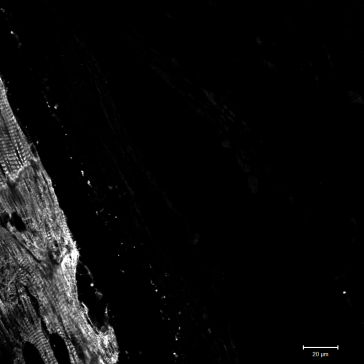 | 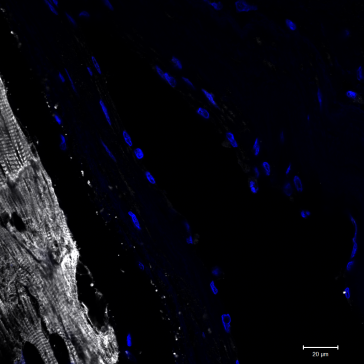 |

**Supplemental Figure 3.** *IL-33 and ST2 protein in human heart.*

Confocal immunofluorescence images of a blood vessel in human heart tissue. Single staining for IL-33 (A), ST2 (B) and troponin (C) in subsequent sections presenting the same region of the heart. Arrow (A, merge) shows IL-33 expression in white blood cell. Original magnification x630. Scale bar=20μm.

|  | DAPI | ST2 | SMA | merge |
| --- | --- | --- | --- | --- |
| A | 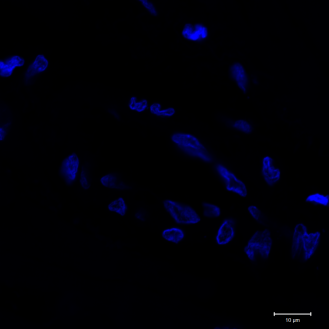 | 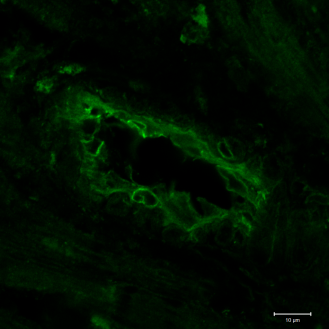 | 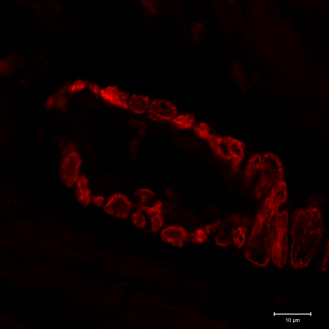 | 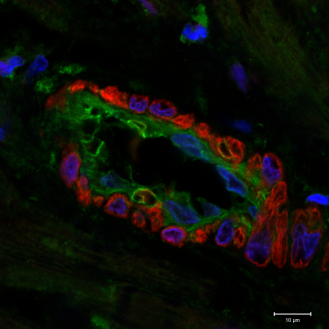 |
|  | DAPI | IL-33 | merge |  |
| B | 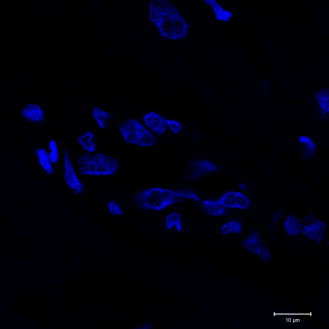 | 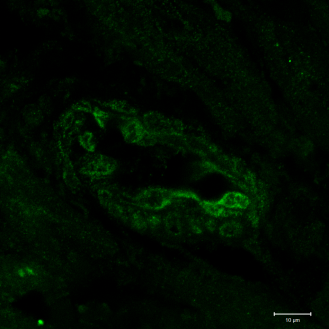 | 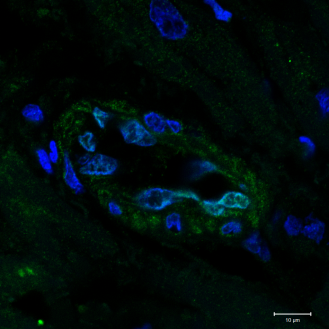 |  |

**Supplemental Figure 4.** *Presence of IL-33 and absence of ST2 protein in smooth muscle cells in human heart.*

Confocal immunofluorescence images of heart tissue. Co-staining of ST2 and smooth muscle actin (SMA, A) or single staining of IL-33 (B) in serial sections representing the same vessel. Original magnification x630. Scale bar=10μm.

|  | DAPI | IL-33 | vWF | Troponin | merge |
| --- | --- | --- | --- | --- | --- |
| B | **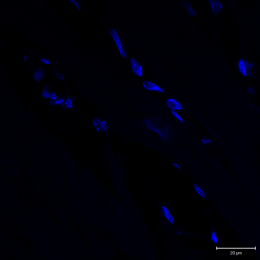** | **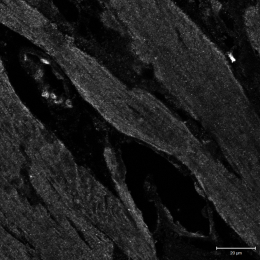** | **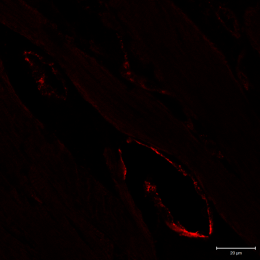** | **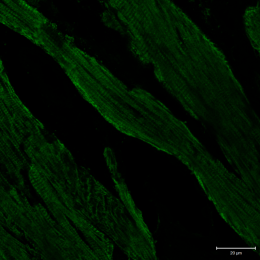** | **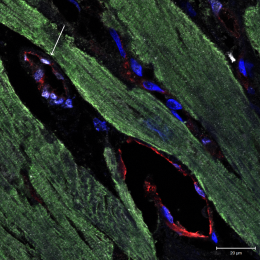** |

**Supplemental Figure 5.** *IL-33 protein in endothelial cells and cardiac myocytes in human heart.*

Confocal immunofluorescence images of heart tissue. Co-staining for IL-33, von Willebrand factor (vWF) and troponin in human heart.Original magnification x630. Scale bar=20μm.
